# The Main Bacterial Communities Identified in the Sites Affected by Periimplantitis: A Systematic Review

**DOI:** 10.3390/microorganisms10061232

**Published:** 2022-06-16

**Authors:** Simina Angela Lăcrimioara Iușan, Ondine Patricia Lucaciu, Nausica Bianca Petrescu, Ioana Codruța Mirică, Dan-Alexandru Toc, Silviu Albu, Carmen Costache

**Affiliations:** 1 Department of Oral Health, Iuliu Hatieganu University of Medicine and Pharmacy, 400012 Cluj-Napoca, Romania; siminaiusan@yahoo.com (S.A.L.I.); nausica_petrescu@yahoo.com (N.B.P.); mirica_codruta@yahoo.com (I.C.M.); 2 Department of Microbiology, Iuliu Hatieganu University of Medicine and Pharmacy, 400012 Cluj-Napoca, Romania; dan_alext@yahoo.com (D.-A.T.); carmen_costache@yahoo.com (C.C.); 3 II-nd Department of Otolaryngology, Iuliu Hatieganu University of Medicine and Pharmacy, 400012 Cluj-Napoca, Romania; silviualbu63@gmail.com

**Keywords:** periimplantitis, dental implants, bacteria, bacterial species, bacterial strains, microorganisms

## Abstract

(1) Background: Periimplantitis is an infectious condition that affects the periimplant tissue and is of bacterial etiology. However, to date, the exact bacterial flora involved in its occurrence is not known. The aim of this literature review was to summarize the articles published on this topic and to identify the main bacterial species isolated in periimplantitis. (2) Methods: The articles published in three databases were researched: Pubmed, Embase and Web of Science using Prisma guides and combinations of MeSH terms. We selected 25 items from the 980 found by applying the inclusion and exclusion criteria. (3) Results: We quantified the results of the 25 studies included in this review. In general, the most commonly identified bacterial species were Gram-negative anaerobic species, as *Prevotella*, *Streptococcus*, *Fusobacterium* and *Treponema*. (4) Conclusion: The most frequent bacteria in the periimplantitis sites identified in this review are Gram-negative anaerobic species, also involved in the pathogenesis of the periodontal disease.

## 1. Introduction

The loss of dental units due to increased life expectancy, aging, lack of regular check-ups at the dentist, poor oral hygiene or various accidents leads to the installation of edentulousness. Once edentation appears, the functionality of the dento-maxillary apparatus, the homeostasis of the alveolar bone, as well as the balance of the digestive and nervous system are affected by the psychological and masticatory implications of tooth loss.

In recent years, the concern among dental practitioners for finding prosthetic rehabilitation solutions as functional as possible and for preserving the remaining dental structures has led to the widespread use of dental implants as a treatment solution of edentulous patients. There are several types of biomaterials from which dental implants can be made, the most used being titanium and titanium alloys, materials with high biocompatibility, high success rates over long periods of follow-up and good mechanical properties [1]. Although titanium implants are widely used, they also have drawbacks such as the gray color that can be visible in the frontal area with thin gingival tissue, which is why more aesthetic alternatives have been found. One of these is the use of Zirconia for dental implants, a material that has aesthetic properties as translucency, the color similar to natural teeth and biocompatibility with the surrounding soft and hard tissues [1,2,3]. The mechanical properties of Zirconia are encouraging, as it has high flexural strength, increased resistance to fracture and corrosion, as well as a low thermal conductivity [3]. Regarding osseointegration, most studies show that the two materials have similar properties [3,4]. Colonization of the surface of zirconium implants by pathogenic bacteria has been shown to be lower compared to implants made of titanium alloys, leading to optimal healing and a low rate of infectious complications [1,3,4].

Regarding the prosthetic rehabilitation on implants, several comparative studies were performed between the dental prostheses made of metal-ceramic and those of zirconia-ceramic, following the main causes of failure. Current information in the literature on the success of monolithic zirconia rehabilitation is limited. According to the studies, the most common causes of failure of crowns and bridges on implants are the fracture of materials, more common in the case of zirconia, either the fracture of the coating ceramics or the fracture of the zirconia structure. Biological complications such as periimplantitis are also more common in zirconia-ceramics than in metalo-ceramics [5,6].

Dental implants have improved oral rehabilitation in edentulous patients and have reported increased success rates over periods of time larger than 10 years [7,8]. However, once inserted into the oral cavity, the implant surfaces are rapidly colonized by commensal bacteria. Bacterial adhesion begins about 30 min after implantation, and after about one to three months the subgingival bacterial community is similar to those around natural teeth [9]. Once dental implants appeared as a new treatment alternative in dentistry, periimplant infections developed as new oral pathologies [10]. Periimplant infections are classified as perimucositis and periimplantitis. If in perimucositis the induced inflammation is limited to the soft tissues, in the case of periimplantitis the lesion progresses to the bone, causing osteolysis [10]. Both diseases have a bacterial etiology, being strongly correlated with the presence of bacterial biofilm, especially of Gram-negative anaerobic bacteria [11], their establishment occurring once the balance between bacterial and host communities is disturbed. Around healthy implants there are frequently found microorganisms from yellow and purple complexes (*Streptococcus*, *Streptococcus oralis*, *Streptococcus mutans*, *Veillonella parvula* and *Actinomyces odontolyticus*, etc.) [9]. Gram negative bacteria including *Prevotella intermedia*, *Porphyromonas gingivalis*, *Prevotella nigrescens*, and *Campylobacter rectus* are rarely found in healthy periimplantar sites. On the contrary, in case of periimplantitis, the main components of the bacterial community are represented by Gram-negative anaerobic bacteria. There are also some cases of periimplantitis induced by Gram-negative aerobic bacteria, but the main etiological factor is represented by Gram-negative anaerobic bacteria [9]. These bacteria communities include *Porphyromonas gingivalis*, *Prevotella intermedia*, *Treponema denticola*, *Tannerella forsythia*, and *Fusobacterium nucleatum*. In addition, the presence of *fungi*, e.g., *Candida albicans* and other microorganisms, such as *Aggregatibacter actinomycetemcomitans* and *Staphylococcus aureus*, has also been reported in places affected by periimplantitis [11]. Previous studies show that *Staphylococcus aureus* adheres around implant components in the first year after implantation and this bacteria has a major role in the development of periimplantitis. *Staphylococcus aureus* appears to play a predominant role in the development of periimplantitis. Dental implants that were affected by periimplantitis were found to be associated with low antibody titer and elevated *Staphylococus aureus* levels. In vitro studies have demonstrated that there is an increased affinity between *Staphylococus aureus* and titanium surfaces [12,13]. With the emergence of a new disease, COVID-19, caused by the infection with SARS-CoV-2, the possible link between this viral infection and periimplantitis has been studied. It is shown that SARS-CoV-2 is present in the saliva and crevicular fluid of patients with COVID-19 [14,15]. Patients with periodontal or periimplant diseases, such as periimplantitis, are at an increased risk of developing COVID-19-associated complications, and patients with COVID-19 risk worsening periodontal or periimplant lesions [16]. During the pandemic, due to the transmission of SARS-CoV-2 through aerosols, dental offices adapted to the new global situation, so that the access of patients suffering from chronic diseases, such as periimplantitis, to dental treatments was limited, this aggravating their condition [14]. At the same time, the increased level of stress and depression in the population, accentuated by the pandemic, caused the aggravation of periodontal diseases by increasing plaque accumulation and gingival bleeding due to neglect of oral hygiene, lack of regular visits to the dental office, as well as by decreasing the healing capacity of the tissues and affecting the body’s response to treatment [14,16,17]. Patients with periodontal disease or periimplantitis have a higher risk of developing complications associated with COVID-19, studies suggesting that lesions in the periodontal and periimplant pockets are an entry point for the virus into the general circulation [15,16]. Certain constituents of plaque in the periimplant sulcus, such as Treponema denticola, Porphyromonas gingivalis or Candida, release proteases that degrade the basement membrane facilitating viral and bacterial invasion [15]. Periodontal and periimplant pockets, rich in aggressive pathogens, can also be a starting point for microorganisms that can be aspirated by people infected with SARS-CoV-2, leading to various complications [16].

The information in the literature about the microbiota in periimplantitis is inaccurate and it is not specified exactly which bacteria are associated with periimplantitis. Recent data suggests that periimplantitis is a polymicrobial anaerobic infection that does not fully correspond to the severity of the disease [12].

The bacterial community located in the oral cavity is diverse, and information on microorganisms involved in the development of periimplant infectious diseases as well as standardized treatment protocols for these diseases are limited. This literature review aimed to highlight the main bacterial colonies isolated from periimplant sites affected by periimplantitis by synthesizing information published on this topic in specialized studies.

## 2. Materials and Methods

### 2.1. Search Strategy

The methodological design of this study is in line with the PRISMA 2020 criteria and guidelines [18]. The protocol of the review was registered within the International Prospective Register of Systematic Reviews (PROSPERO) under registration number CRD42022335476. In this study, we address the following question: “Which are the most common bacterial species in periimplantitis?” To answer this question, a systematic literature search was carried out in 3 databases: PubMed, Embase, and Web of Science. The search strategy consisted of different combinations of MeSH keywords: “periimplantitis”, “bacteria”, “biofilm”, “microorganisms”, “microbiota”, “dental implant”: (bacteria) AND (periimplantitis), (biofilm) AND (periimplantitis), (microorganisms) AND (periimplantitis), (microbiota) AND (periimplantitis), (biofilm) AND (dental implant), ((bacteria) AND (dental implant)) AND (periimplantitis), (microbiome) AND (periimplantitis), (“Bacterial strains and periimplantitis” or “biofilms and periimplantitis” or “bacterial cultures and periimplantitis” or “types of bacteria and periimplantitis”), the filters applied being: Clinical Study, Clinical Trial, Randomized Controlled Trial, Other Animals, Humans, in the last 10 years. Two researchers independently performed the database literature search, and subsequently the results were confronted.

### 2.2. Inclusion and Exclusion Criteria

The inclusion criteria were the articles published in the three databases mentioned above, in the last ten years, between January 2012 until March 2022. We selected studies performed on patients with dental implants suffering from periimplantitis and those in which samples of bacterial plaque were collected from periimplant sulcus affected by periimplantitis, analyzed and provided results on the microbial flora involved in periimplantitis.

Among the exclusion criteria were: articles published in a language other than English or French, articles other than those mentioned above, such as systematic reviews or meta-analyzes, experimental or in vitro studies.

### 2.3. Study Selection and Data Collection

Initially, the titles and abstracts of the selected articles were checked for the relevance of the topic, in relation to the proposed research. Subsequently, a full text analysis of all eligible articles was performed based on the inclusion and exclusion criteria. All articles that met the eligibility criteria were selected, and a standardized document was used to collect information on authors, year of publication, bacterial isolation methods and bacteria identified from these studies. Initially, 980 items from the 3 databases were selected according to the search terms mentioned above. Subsequently, after the elimination of duplicates, 201 articles remained, which were analyzed in detail based on inclusion and exclusion criteria. Finally, 25 articles were selected that met the eligibility criteria and introduced in this review. The selected articles were analyzed and classified according to the methods of isolation and identification of bacterial species. To collect data from selected articles, a table was created that included information on the authors of the articles, the year of publication, the study design, population characteristics, the isolation techniques and microbiological methods of bacterial identification, as well as the main bacterial species identified in each study (Table 1).

The quality assessment of the included studies in this review was performed using the Newcastle-Ottawa Scale (NOS) [42]. The NOS scale quantifies three quality parameters (selection, comparability and outcome). These parameters are divided into eight specific categories. Each item on the scale is scored with a maximum of one point, except for comparability, which can be scored with up to two points. The maximum score that can be obtained for each study is 9 [43]. Studies with NOS scores 0–3 were considered as low quality, 4–6 moderate quality and 7–9 were considered as high quality (Table 2).

## 3. Results

### 3.1. Bibliographic Documentation and Selection of Articles

**Figure 1:**
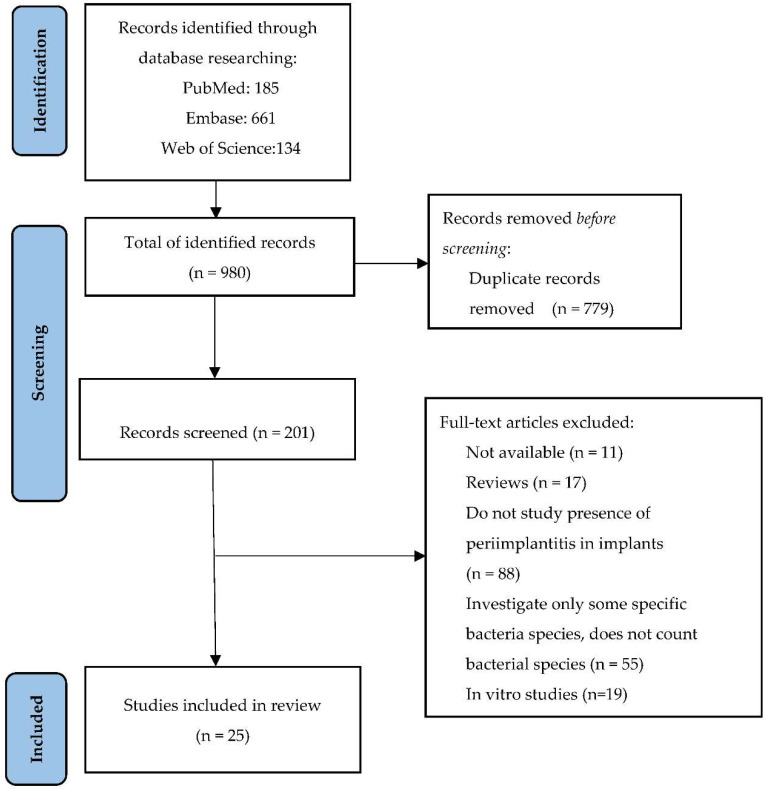
PRISMA flow diagram. Study selection for review.

The process of searching and selecting the articles included in this literature review according to the PRISMA requirements is presented in Figure 1. Initially, 980 articles were selected from the three databases: PubMed, Embase and Web of Science that matched the search terms. Out of these, 779 duplicates were identified and removed. The remaining 201 articles were checked, 11 of them being removed because they were not available in full text, another 17 being literature reviews and 19 being in vitro studies. Other elimination criteria were the evaluation of specific bacteria, the lack of quantitative analysis of bacterial species or the absence of periimplantitis. Finally, after a detailed analysis, 25 articles that analyze the bacterial flora involved in periimplantitis were included in this study (Figure 1).

### 3.2. Clinical and Microbiological Characteristics of the Included Studies

Table 1 summarizes the most relevant information in the included studies, such as title, authors, year of publication, study design, population characteristics, methods of isolation and bacterial identification, and the main bacterial species identified. Out of a total of 25 articles, six of them used classical bacterial culture methods to identify microorganisms involved in the development of periimplantitis and 17 used modern molecular biology techniques, DNA-DNA hybridization or RNA sequencing, and two of them used combined bacterial culture and DNA hybridization techniques.

Quality assessment of the included studies in this review performed using NOS Scale emphasized that 16 studies were of high quality, eight studies were of moderate quality and one study was of low quality. The results are summarized in Table 2. The average NOS score for the quality assessment of the included studies in this review was 6.6.

A classification of the microorganisms identified in the 25 studies included in this review was made, and the frequency with which each microorganism was found in them was subsequently quantified. In addition, a separate classification was performed on studies that used the bacterial culture technique to identify microorganisms, analyzing the differences between the two classifications.

In general, the most commonly identified bacterial species were *Prevotella*, and *Streptococcus*, Gram-negative anaerobic species. *Prevotella* was found in 17 of the 25 studies, so with a frequency of 68%. In this bacterial species, *Prevotella intermedia* was isolated in 7 of the 17 studies (41.17%), *Prevotella nigrescens* in 4 of 17 (23.52%), *Prevotella fusca*, *Prevotella multiformis* and *Prevotella denticola*, in one of the 17 articles (5.88%).

*Streptococcus* was found in 17 of the 25 studies, so with a frequency of 68%. The following bacterial species were identified from the genus *Streptococcus: Streptococcus oralis* and *Streptococcus constellatus* in 5 out of 17 studies (29.41%), *Streptococcus mitis*, *Streptococcus intermedius* in 4 out of 17 studies (23.52%), *Streptococcus gordonii* in 3 out of 17 studies (17.64%), *Streptococcus sanguinis*, *Streptococcus salivarius*, *Streptococcus pneumoniae*, *Streptococcus anginosus* in 1 out of 17 studies (5.88%).

The genera *Fusobacterium* were isolated in 16 out of the 25 studies, so with a frequency of 64%. Of the genus *Fusobacterium*, *Fusobacterium nucleatum* was isolated in 8 of 16 cases, respectively, 50%.

The fourth most common bacterial genus was *Treponema* in 15 of the 25 studies, 60%, respectively. *Treponema denticola* was isolated from this species, 5 out of 15 studies (33.33%), *Treponema socranski* and *Treponema lecithinolyticum*, 2 out of 15 studies (13.33%), *Treponema parvum* and *Treponema maltophilum* in 1 out of 15 studies (6.66%).

The genus *Porphyromonas*, identified in 14 of the 25 studies (56%), includes the species *Porphyromonas gingivalis*, isolated in 10 of 14 studies (71.42%), respectively, *Porphyromonas endodontalis*, 3 of 14 studies (21.42%).

*Tannerella* was identified in 11 of the 25 studies (44%), with *Tannerella* *forsythia* being isolated in 9 of 11 (81.81%).

The genus *Neisseria*, identified in 9 of the 25 studies (36%), the species *Neisseria flavescens* being found in 2 of the 9 studies (22.22%).

*Campylobacter*, also identified in 9 of the 25 studies (36%), includes *Campylobacter rectus*, 5 out of 9 (55.55%), *Campylobacter gracilis*, 4 out of 9 (44.44%), *Campylobacter concisus*, 2 of 9 (22.22%) and *Campylobacter showae* 1 of 9 (11.11%).

The genus *Fretibacterium*, also identified in 7 of the 25 studies (28%), includes the species *Fretibacterium fastidiosum*, isolated in 4 of the 7 studies (57.14%).

*Filifactor*, isolated in 7 of the 25 studies (28%), includes the species *Filifactor alocis*, identified in 6 of the 7 studies (85.71%). *Capnocytophaga* was also isolated in 7 of the 25 studies (28%).

The species *Parvimonas micra*, genus *Parvimonas*, was identified in 6 of the 25 studies, respectively 24%.

*Veillonella*, identified in 5 of the 25 studies (20%), includes the species *Veillonella parvula*, identified in 3 of the 5 studies (60%).

The genus *Peptostreptococcus*, identified in 4 of the 25 studies (16%), includes the species *Peptostreptococcus asaccharolyticus*, isolated in 1 of the 4 studies (25%), respectively, *Peptostreptococcus micros* in one of the 4 studies (25%).

*Haemophilus*, identified in 5 of the 25 studies (20%), includes the species *Haemophilus parainfluenzae*, identified in 2 of the 5 studies (40%), respectively, *Haemophilus influenzae* in one of the 5 studies (20%).

The genus *Eubacterium*, identified in 5 out of 25 studies (20%), includes the species *Eubacterium infirmum*, 2 out of 5 studies (40%), respectively, *Eubacterium minutum*, 1 out of 5 studies (20%).

The genus *Actinomyces* includes the species *Actinomyces odontolyticus*, isolated in 4 of the 25 studies (16%), *Actinomyces naeslundii*, in 2 of the 25 studies (8%), respectively, *Actinomyces massiliensis*, *Actinomyces johnsonii* and *Actinomyces cardiffensis* in one of the 25 studies (4%).

*Staphylococcus*, identified in 4 of the 25 studies (16%), includes the species *Staphylococcus aureus*, identified in 3 of 4 studies (75%), *Staphylococcus Anaerobius*, in 2 of 4 studies (50%), respectively, *Staphylococcus epidermidis*, 1 of 4 studies (25%).

The genus *Leptotrichia*, identified in 4 of the 25 studies (16%), includes the species *Leptotrichia hofstadii* and *Leptotrichia buccalis* identified in one of the 25 studies (4%).

*Alloprevotella*, identified in 4 of the 25 studies (16%) includes the species *Alloprevotella tannarae*, identified in one of the 4 studies (25%).

Other bacterial species were sporadically identified in one or two studies out of 25, 4% and 8%, respectively. These include *Synergistetes*, *Rothia aeria*, *Lautropia mirabilis*, *Johnsonella*, *Gemella*, *A. Actinomycetemcomitans*, *Dialister invisus*, *Eikenella Corodens*, etc.

Regarding the species identified by microbial cultures, 6 of the 25 studies that used this method to isolate bacteria from periimplant sites were identified. The most common were the genera *Veillonella*, *Prevotella* and *Fusobacterium*, with a frequency of 3 out of 6 studies, respectively, 50%. *Prevotella intermedia* was identified in 1 of 3 studies (33.33%), *Prevotella oralis* in 1 of 3 (33.33%) and *Fusobacterium nucleatum* in 2 of 3 studies (66.66%).

*Actinomyces odontolyticus*, genus *Actinomyces* was isolated in 2 of 6 studies, respectively, 33.33%, and *Actinomyces naeslundii* in one of 6 studies, respectively, 16.66%.

*Porphyromonas*, identified in 3 of 6 studies, 50%, with the species *Porphyromonas gingivalis*, isolated in 2 of the 3 studies, 66.66%.

*Streptococcus oralis*, *Streptococcus constellatus*, *Streptococcus intermedius* identified in 3 of 6 studies (50%). The species *Streptococcus mitis*, *Neisseria flavescens* isolated in 2 out of 6 studies, respectively, 33.33%. The genus *Peptostreptococcus* spp., the species *Peptostreptococcus asaccharolyticus* in 1 out of 6 studies (16.66%).

Other species were sporadically identified in only one of the 6 studies (16.66%) in which the bacterial culture technique was used: *Tannerella*, *Streptococcus anginosus*, *Staphylococcus epidermidis*, *Streptococcus salivarius*, *Enterococcus faecalis*, *Candida albicans Pseudomonas aeruginosa*, *Streptococcus*, *Rothia mucilaginosa*, *Rothia aeria*, *Haemophilus parainfluenzae*, *Klebsiella pneumoniae*, *Klebsiella oxytoca*, *Veilonella parvula*, *Actinomyces naeslundii*, *Capnocytophaga* spp., *Prevotella oralis*, *S. Gordonii*, *Slackia exigua*, *Gemella morbillorum.*

## 4. Discussion

The complications related to dental implants are mainly inflammatory lesions of the bone and soft tissues surrounding the implants and their restorative components, induced by the accumulation of bacterial biofilm [8,44]. Periimplantitis is defined as an inflammatory disease of the mucosa surrounding an implant accompanied by progressive loss of periimplant support bone [44]. It is generally perceived that after implant insertion and initial loading, during the healing process, between 0.5 and 2 mm of crestal bone height is lost. Any radiographic evidence of additional bone loss suggests a periimplant condition [8]. Periimplantitis has an infectious etiology, and the diversity of bacteria in the oral cavity is increased. However, information on the microbiota involved in periimplantitis is limited and it is unclear whether there is a specific group of bacteria that may be associated with periimplantitis. Through this review, we wanted to summarize the results obtained in specialized studies, in order to identify the main pathogens involved in the development of periimplantitis. The main species identified in most studies included in this literature review are Gram-negative anaerobic bacteria such as those in the red complex, a highly virulent complex containing particularly aggressive species as *Porphyromonas gingivalis*, *Tannerella forsythia* and *Treponema denticola*. These bacteria are also correlated with the loss of the gingivo-periodontal junction in periodontal diseases. At the same time, bacteria from the orange complex, *Prevotella intermedia*, *Fusobacterium nucleatum*, *Campylobacter*, *Streptococcus constellatus*, *Peptostreptococcus* and *Eubacterium* were identified in most studies in this review. These bacteria have the ability to make connections and enhance the destructive effect of bacteria in the red complex by promoting their colonization. These findings are consistent with other studies published in the literature [9,11], which also show that the main components of the bacterial community are Gram-negative anaerobic bacteria, as confirmed in this study. Perimucositis and periimplantitis have similar characteristics to gingivitis and parodontitis, that occur around natural dentition. These diseases have multifactorial etiologies and are strongly correlated with bacterial biofilm, especially with Gram-negative anaerobic bacteria [11]. Numerous studies have found the presence of a high number of periodontopathogenic bacteria of the red complex and those in the orange complex in the sites affected by periimplantitis and have highlighted the correlations between the two conditions and how they influence each other when coexisting within the same oral cavity [9,10,12,13,45].

In addition to Gram-negative anaerobic bacteria and those in the red and orange complexes, a high number of studies included in this review discovered Gram-negativeaerobic species such as *Neisseria*, yellow and purple complex species: *Streptococcus*, *Veillonella*, and Gram-positive anaerobic species: *Filifactor*, *Parvimonas micra.*

Therefore, the most common bacteria in the periimplantitis sites identified in this review are Gram-negative anaerobic species, also involved in the pathogenesis of the periodontal disease. This aspect is particularly important to establish new preventive measures and new therapeutic protocols, in order to reduce the incidence of this condition among patients.

The treatment of periimplant diseases is complex, the management of these diseases being difficult to achieve. The study of the bacterial flora involved in periimplantitis and the knowledge of the microorganisms incriminated in the pathogenesis of this disease can be a starting point in the research of new therapeutic means in order to achieve a personalized treatment targeted on the main periimplant pathogens. New preventive measures may also be developed in order to reduce the incidence of these conditions in patients or to prevent recurrence after treatment. All the studies included in this review were assessed for risk of bias with NOS Scale Tool [42], independently by two authors. Divergences in the assessment were solved by discussion and by re-evaluating the article. According to NOS Scale there are three groups of risk of bias: low risk of bias (7–9 NOS scores), high risk of bias (4–6 NOS scores) and very high risk of bias (0–3 NOS scores) [46]. The majority of the studies included in this review are at low risk of bias and only one study is considered at very high risk of bias.

The main limitation of this review is the relatively heterogeneity of the studies included in this research. The included articles have different study design, different methods of identifying the clusters of bacteria involved in periimplantitis and the studies are performed on different types of implants. Additionally, the results of counting bacteria are reported in different measurements units. Analysis of the differences in bacterial colonization regarding the type, material and surface design of the implants was impossible because this information was not available in all the articles included in this review.

## 5. Conclusions

Summarizing the results obtained in this review, we can state that there is a correlation between the germs involved in the pathogenesis of periodontal diseases and those that cause periimplant diseases. The bacteria identified in most of the analyzed studies: *Prevotella*, *Streptococcus*, *Fusobacterium*, *Treponema*, *Tannerella*, and *Porphyromonas gingivalis* are mostly anaerobic bacteria, pathogens with high virulence, also involved in the development of periodontal diseases, which require special treatment protocols.

## Figures and Tables

**Table 1:** Studies included in this review and the main bacterial species identified in each study.

Article Title	Authors	Year of Publication	Study Design	Study Samples	Population Characteristics	Bacterial Isolation Technique	Isolated Bacteria
Diversity analysis of subgingival microbial bacteria in periimplantitis in Uygur population	Gao et al. [9]	2018	Observational Study	40 samples of gingival crevicular fluid divided into two groups: healthy implants (Control group) and periimplantitis (Case group)	Uygur patients who had treatment with dental implants from 2013 to 2016	DNA extraction, PCR amplification and 16S rRNA gene sequencing	*Prevotella*, *Streptococcus*, *Acinetobacter*, *Fusobacterium*, *Neisseria*, *Porphyromonas*, *Treponema*, *Leptothrix*, *Capnocytophaga*
Efficiency of photodynamic therapy in the treatment of periimplantitis—A three-month randomized controlled clinical trial	Rakašević et al. [19]	2016	Randomized controlled clinical trial	Samples from 52 periimplantitis sites divided into two groups (Study group and Control group)	Patients with periimplantitis who presented in two dental clinics in Belgrade between January 2014 until February 2015	Bacterial culture for the diagnosis of aerobic and anaerobic pathogens	*Veillonella* spp., *Prevotella intermedia*, *Peptostreptococcus* spp., *Peptostreptococcus asaccharolyticus*, *Porphyromonas gingivalis*, *Fusobacterium nucleatum*, *Actinomyces odontolyticus*
Bacterial profiles and proteolytic activity in periimplantitis versus healthy sites	Neilands et al. [20]	2015	Non-randomized, controlled, clinical study	50 samples (25 from healthy subjects, 25 from periimplantitis sites)	Patients with dental implants treatment in the past, attending maintenance appointments in a dental clinic in Sweden	Bacterial culture on Brucella agar	*Porphyromonas/Prevotella*, *Fusobacterium*, *Tannerella*, *Streptococcus oralis*, *Streptococcus mitis*, *Streptococcus anginosus*, *Streptococcus constellatus*, *Streptococcus intermedius*
Short-term effects of hyaluronic acid on the subgingival microbiome in periimplantitis: A randomized controlled clinical trial	Soriano-Lerma et al. [21]	2020	Randomized controlled trial	108 samples divided into 3 groups (Test group, Control group 1, Control group 2)	Patients diagnosticated with periimplantitis in a private dental office in Spain	DNA isolation, PCR amplification and 16S rRNA gene sequencing	*Fusobacterium*, *Prevotella*, *Porphyromonas*, *Ralstonia*, *Sphingomonas*, *Streptococcus*, *Treponema*, *Propionibacterium*, *Alloprevotella*, *Veillonella*, *Lactobacillus*, *Haemophilus*, *Staphylococcus*, *Campylobacter*, *Tannerella*
A randomized clinical trial of an adjunct diode laser application for the nonsurgical treatment of periimplantitis	Arısan et al. [22]	2015	Randomized clinical trial	Samples collected from 24 implants affected by periimplantitis at baseline and 1 month after intervention	10 patients diagnosticated with periimplantitis who went to the department clinic in Istanbul University between February 2010 and May 2013	DNA extraction, PCR amplification and hybridization procedures	*Actinomyces odontolyticus*, *Actinomyces viscosus*, *Aggregatibacteractinomycetemcomitans*, *Campylobacter concisus*, *Campylobacter gracilis*, *Campylobacterrectus/showae*, *Capnocytophaga gingivalis/sputigena/ochracea*, *Eikenella corrodens*, *Eubacterium nodatum*, *Fusobacterium nucleatum*, *Peptostreptococcus micros*, *Porphyromonas gingivalis*, *Prevotella intermedia*, *Prevotellanigrescens*, *Streptococcus constellatus group*, *Streptococcusgordonii group*, *Streptococcus mitis group*, *Tannerella forsythia (Bacteroides forsythus; Tannerella forsythensis)*, *Treponema denticola*, *and Veillonella parvula.*
The effects of Lactobacillus reuteri probiotics combined with azithromycin on periimplantitis: A randomized placebo-controlled study	Tada et al. [23]	2017	Randomized placebo-controlled study	Samples collected from periimplantitis sites at baseline and 4, 12, and 24 weeks after allocated treatment	30 patients diagnosticated with periimplantitis from 7 different institutions including Kyushu Dental University Hospital, Japan, divided into 2 groups, placebo and probiotics	DNA isolation, PCR amplification and 16S rRNA gene sequencing	*Treponema denticola*, *Fusobacterium nucleatum*, *Peptostreptococcus micros*, *Streptococcus constellatus*, *Prevotella nigrescens*, *Tannerella forsythia*, *Campylobacter gracilis. Prevotella intermedia*, *Campylobacter rectus*, *Porphyromonas gingivalis*, *Veillonella parvula*, *Streptococcus gordonii*, *Capnocytophaga*, *Streptococcus mitis*
Effectiveness of enamel matrix derivative on the clinical and microbiological outcomes following surgical regenerativetreatment of periimplantitis. A randomized controlled trial	Isehed et al. [24]	2016	Randomized controlled trial	Samples collected from the deepest pocket of the each implant at baseline, 2 weeks, 3, 6, and 12 months after surgery treatment	29 patients diagnosticated with periimplantitis from a periodontology clinic in Sweden	DNA extraction with Gen Elute Bacterial Geno-mic DNA kit (Sigma Aldrich, St. Louis, MO, USA), bacterial characterization by the HOMIM microarray	*Fusobacteria (cluster probe)*, *Parvimonas micra*, *Porphyromonas* sp., *Eubacterium nodatum*, *Porphyromonas gingivalis*, *Ochrobactrum anthropi*, *Tannerella forsythia* and *Campylobacter concisus/Campylobacter rectus.*
Adjunctive Systemic and Local Antimicrobial Therapy in the Surgical Treatment of Periimplantitis: A Randomized Controlled Clinical Trial	Carcuac et al. [25]	2016	Randomized Controlled Clinical Trial	Samples collected from periimplantitis sites at baseline, 3, 6 and 12 months after surgery	100 patients with severe periimplantitis who were referred to 2 clinics specialized in periodontics in Sweden	Culture and checkerboard DNA-DNA hybridization analyses	*Fusobacterium nucleatum*, *Prevotella intermedia/Prevotella nigrescens*, *Campylobacter rectus*, *Porphyromonas gingivalis*, *Tannerella forsythia*, *Porphyromonas endodontalis*, *Parvimonas micra*
Comparison of the effects of air-powder abrasion, chemical decontamination, or their combination in open-flap surface decontamination of implants failed for periimplantitis: an ex vivo study	Pranno et al. [26]	2021	Single-blind, randomized, controlled, ex vivo study	80 samples collected from the retrieved implants	20 patients from Oral Surgery Unit University of Rome with minimum 4 implants affected by periimplantitis which need to be explanted	Bacterial culture techniques: for aerobic bacteria—Columbia sheep blood agar plates and for anaerobic—Schaedler sheep blood agar	*Staphylococcus aureus*, *Streptococcus mitis/oralis*, *Staphylococcus epidermidis* and *Streptococcus salivarius. Enterococcus faecalis*, *Candida albicans*, *Pseudomonas aeruginosa* and *Neisseria flavescens*
The Efficacy of a Diode Laser on Titanium Implants for the Reduction of Microorganisms That Cause Periimplantitis	Wawrzyk et al. [27]	2021	Clinical study	Samples collected from saliva, the surfaces of the crowns and dental implants components	3 patients with advanced periimplantitis	Bacterial culture technique of anaerobic using Schaedler horse blood agar	*Staphylococcus aureus*, *Streptococcus constellatus*, *Streptococcus oralis*, *Streptococcus pneumoniae*, *Rothia mucilaginosa*, *and Rothia aeria*, *and the following Gram-negative bacteria: Haemophilus parainfluenzae*, *Klebsiella pneumoniae*, *Klebsiella oxytoca*, *and Veilonella parvula. Candida guilliermondii*, *Actinomyces odontolyticus*
Investigation of antibiotic susceptibility of the bacterial isolates and local flora changes after complex therapy in chronic periodontitis and periimplantitis	Ciobanu et al. [28]	2018	Clinical study	Samples collected from sites with periimplantitis before and after therapy	Patients diagnosticated with chronic periimplantitis	Culture examination	*Capnocytophaga* spp., *Prevotella oralis*, *S. intermedius*, *S. gordonii*, *Veillonella* spp.
Shift of microbial composition of periimplantitis-associated oral biofilm as revealed by 16S rRNA gene cloning	Al-Ahmad et al. [29]	2018	Cross-sectional study	Samples collected from the deepest sites of periimplantitis and from the periimplantar healthy sulcus	10 patients with at least one implant affected by periimplantitis and one healthy implant	DNA extraction and PCR amplification of 16S rRNA genes	*Streptococcus* spp., *Prevotella* spp., *Fusobacterium* spp., *Eubacterium* spp., *Porphyromonas gingivalis*, *Treponema* spp., *Campylobacter* spp., *Filifactor alocis*, *Abitrophia defectiva*, *Alloprevotella tannarae*, *Neisseria* spp., *Parvimonas micra*, *Selenomonas* spp., *Capnocytophaga* spp., *Atopobium* spp., *Peptostreptococcus* spp., *Tannerella forsythia*, *Scadovia wiggisiae*, *Bacteroidetes bacterium*, *Eikenella Corodens*, *Fretibacterium fastidiosum*, *Johnsonella ignava*, *Synergistales bacterium*, *Dialister invisus*, *Raoultella* sp.
Subgingival microbiome in patients with healthy and ailing dental implants	Zheng et al. [30]	2015	Clinical study	Samples collected from periimplantar sulcus and pockets	10 patients with healthy implants, 8 patients with perimucositis and 6 with periimplantitis	Microbial DNA extraction, 16S rRNA gene library preparation, and pyrosequencing	*Leptotrichia hofstadii*, *Eubacterium infirmum*, *Kingella denitrificans*, *Actinomycescardiffensis*, *Eubacterium minutum*, *Treponema lecithinolyticum*, *and Gemella sanguinis were higher in PI sites Streptococcus*, *Leptotrichia*, *Actinomyces*, *Capnocytophaga*, *Prevotella*, *Fusobacterium*, *Neisseria*
Microbiota in Gingival Crevicular Fluid Before and After Mechanical Debridement With Antimicrobial Photodynamic Therapy in Periimplantitis	Wang et al. [31]	2022	Clinical study	61 samples collected from all the implants: before treatment and 7, 14, 30, 60 and 180 days after treatment	9 patients presented at Department of Stomatology in Beijing Hospital with 14 implants affected by periimplantitis	Bacterial 16S rRNA was amplified and sequenced using an Illumina MiSeq platform	*Bacteroidetes*, *Proteobacteria*, *Firmicutes*, *Fusobacteria*, *Spirochaetes*, *Synergistetes*, and *Actinobacteria Prevotella*, *Neisseria*, *Fusobacterium*, *Porphyromonas*, *Treponema*, *Streptococcus*, *Haemophilus*, *Capnocytophaga*, *Leptotrichia*, and *Fretibacterium*
Microbiological findings in early and late implant loss: an observational clinical case-controlled study	Korsch et al. [32]	2021	Observational clinical case–control study	Samples collected from implants affected by severe periimplantitis without any chance of preservation and from healthy implants as controls	48 patients with 53 implants were introduced in the study	DNA extraction, PCR amplification and 16S rRNA gene sequencing	*Treponema* sp., *Streptococcus*, *Fretibacterium*, *Anaerovoracaceae uncl*, *Desulfobulbus* sp., *Pseudoramibacter alactolyticus*, *Dialister pneumosintes*, *Streptococcus sanguinis*, *Shewanella* sp., *Pantoea* sp., *Haemophilus* sp., *Haemophilus parainfluenzae*, *Pseudomonas* sp., *Lautropia mirabilis*, *Actinomyces naeslundii*
Strong oral plaque microbiome signatures for dental implant diseases identified by strain-resolution metagenomics	Ghensi et al. [33]	2020	Clinical study	Samples collected from each implant and from contralateral healthy implant or tooth for every patient included in the study	80 patients enrolled in the study: 28 with healthy implants, 28 with mucositis and 24 with periimplantitis	DNA extraction	*P. gingivalis*, *T. forsythia*, *Treponema denticola*, *P. endodontalis*, *F. fastidiosum*, *Filifactor alocis*, *Desulfobulbus* spp., *T. lecithinolyticum*
Cluster of bacteria associated with periimplantitis	Persson et al. [12]	2014	Retrospective clinical study	Samples collected at one implant with periimplantitis in each of 166 patients and from 47 healthy implants	166 patients with periimplantitis and 47 patients with healthy dental implants	Checkerboard DNA–DNA hybridization	*Actinomyces odontolyticus*, *A. actinomycetemcomitans (a)*, *Campylobacter gracilis*, *Campylobacter rectus*, *Campylobacter showae*, *Helicobacter pylori*, *Haemophilus influenzae*, *Leptothrichia buccalis*, *P. intermedia*, *Propionybacterium acnes*, *Porphyromonas endodontalis*, *P. gingivalis*, *Staph. aureus*, *Staph. anaerobius*, *Streptococcus intermedius*, *Streptococcus mitis*, *T. forsythia*, *T. denticola*, *and Treponema socranskii.*
Microbiological diversity of periimplantitis biofilm by Sanger sequencing	da Silva et al. [34]	2014	Clinical study	Samples collected from the deepest pocket depth in the test group and from mesial site of healthy implants	20 individuals, 10 with healthy implants and 10 with at least one implant with periimplantitis, both groups with minimum 10 periodontally healthy teeth	Extraction of DNA, PCR amplification of universal 16S rRNA	*Fusobacterium nucleatum*, *Campylobacter gracilis*, *Dialister invisus*, *Streptococcus* sp., *Eubacterium infirmum*, *Filifactor alocis and Mitsuokella* sp., *Parvimonas micra* and *Prevotella intermedia*
Analysis of bacterial flora associated with periimplantitis using obligate anaerobic culture technique and 16S rDNA gene sequence	Tamura et al. [35]	2013	Clinical study	Samples collected from the deepest sites of the both groups, test and control	30 patients, 15 diagnosticated with periimplantitis, 15 with healthy implants	Culture technique and 16S rDNA gene sequence	*Streptococcus*, *Eubacterium*, *Prevotella*, *Actinomyces*, *Fusobacterium*, *Eubacterium nodatum*, *Prevotella intermedia*, *Fusobacterium nucleatum*, *Filifactor alocis*, *E brachy*, *Parascardovia denticolenns*, *Parvimonas micra*
Microbial profiles of peri-implant mucositis and periimplantitis: submucosal microbial dysbiosis correlates with disease severity	Shi et al. [36]	2022	Cross-sectional study	Samples collected from 64 patients, 27 with perimucositis and 37 with periimplantitis	Patients with periimplantitis or perimucositis presented in Dep. Of Oral Implantology in Zhejiang University School of Medicine, China	DNA extraction, PCR amplification and 16S rRNA gene sequencing	*Porphyromonas*, *Fusobacterium*, *Treponema and Prevotella*, *Campylobacter*, *Filifactor*, *Alloprevotella*
Exploring the microbiome of healthy and diseased peri-implant sites using Illumina sequencing	Sanz-Martin et al. [37]	2017	Clinical study	Sample collection from 32 healthy implants and from 35 implants affected by periimplantitis	Patients with healthy implants and with periimplantitis presented in center of Dental Medicine at the University of Zürich	Bacterial nucleic acids isolation, sample DNA analyzed by sequencing the 16S rRNA gene V3-V4 hypervariable region	*Porphyromonas (phylum Bacteroidetes)*, *Treponema (phylum Spirochetes)*, *Filifactor (phylum Firmicutes)*, *Fretibacterium (phylum Synergistetes) and Tannerella (phylum Bacteroidetes)*
Intra-oral single-site comparisons of periodontal and peri-implant microbiota in health and disease	Yu et al. [38]	2019	Clinical study	Samples collected from 4 sites for each patient: Healthy implant, healthy tooth, periimplantitis site and periodontitis site	18 Chinese partial dentate patients with both periimplantitis and periodontitis	DNA extraction, PCR amplification	*Bacteroidetes and Prevotella taxa (including P. denticola*, *P. multiformis and P. fusca).*
Identification of microbiota in periimplantitis pockets by matrix assisted laser desorption/ionization time-of-flight mass spectrometry	Yeh et al. [39]	2019	Clinical study	Samples collected from periimplantitis pockets	12 patients with periimplantitis	Culture examination	*Neisseria flavescen*, *Streptococcus constellatus*, *Slackia exigua*, *Streptococcus intermedius*, *Fusobacteriumnucleatum*, *Gemella morbillorum and Gram-positive anaerobic Bacillus*
Intraindividual variation in core microbiota in periimplantitis and periodontitis	Maruyama et al. [40]	2014	Clinical study	Samples collected from the deepest pockets in periimplantitis sites and in periodontitis sites	20 Patients with both periimplantitis and periodontitis	DNA extraction and PCR amplification of 16S rRNA genes	*Olsenella*, *Sphingomonas*, *Peptostreptococcus*, *unclassified Neisseriaceae*, *genus Desulfomicrobium*, *Actinomyces johnsonii*, *Fusobacterium nucleatum*, *Porphyromonas gingivalis*, *Streptococcus oralis*, *Treponema denticola*, *and Treponema socranskii Achromobacter xylosoxidans*, *Actinomyces massiliensis*, *and Porphyromonas* sp.
The severity of human periimplantitislesions correlates with the level of submucosal microbial dysbiosis	Kröger et al. [41]	2018	Cohort study or case–control study	Samples collected from all 45 implants affected by periimplantitis	30 patients with at list one implant with periimplantitis	DNA extraction and PCR amplification of 16S rRNA genes	*Eubacteriaceae*, *Fretibacterium* sp., *Fretibacterium fastidiosum*, *Peptostreptococcaceae*, *Alloprevotella* sp., *Fastidiosipila sanguinis*, *Filifactor alocis*, *Peptostreptococcaceae*, *Bacteriodetes bacterium*, *Treponema parvum*, *Clostridiales bacterium*, and *Orobacterium*, *Granulicatella elegans*, *Rothia aeria*, *Corynebacterium durum*, *Veillonella dispar*, *Acinetobacter*

**Table 2:** NOS scores for the included studies.

Study	Selection	Comparability	Outcome	NOS Score
*Case–control studies*				
Gao et al. [9]	***	*	***	7
Rakašević et al. [19]	***	**	***	8
Neilands et al. [20]	***	*	***	7
Soriano-Lerma et al. [21]	***	*	**	6
Arısan et al. [22]	***	**	***	8
Tada et al. [23]	***	**	***	8
Isehed et al. [24]	***	**	**	7
Carcuac et al. [25]	***	**	**	7
Pranno et al. [26]	***	*	***	7
Wawrzyk et al. [27]	**	*	***	6
Ciobanu et al. [28]	*	*	***	5
Al-Ahmad et al. [29]	**	*	***	6
Zheng et al. [30]	**	*	***	6
Korsch et al. [32]	**	*	***	6
Persson et al. [12]	***	*	***	7
da Silva et al. [34]	***	*	***	7
Tamura et al. [35]	***	*	***	7
Shi et al. [36]	***	*	***	7
Sanz-Martin et al. [37]	***	*	**	6
Yeh et al. [39]	**	*		3
Maruyama et al. [40]	***	*	***	7
Kröger et al. [41]	**	*	*	4
*Cohort studies*				
Wang et al. [31]	***	*	***	7
Ghensi et al. [33]	****	*	***	8
Yu et al. [38]	****	*	***	8

NOS Scale star system for quality assessment of studies.The higher number of stars (*), the better quality. The maximum (*) possible are 9: **** for Selection, ** for Comparability and *** for Outcome.
